# Predicting potential global and future distributions of the African armyworm (*Spodoptera exempta*) using species distribution models

**DOI:** 10.1038/s41598-022-19983-y

**Published:** 2022-09-28

**Authors:** Irene Gómez-Undiano, Francis Musavi, Wilfred L. Mushobozi, Grace M. David, Roger Day, Regan Early, Kenneth Wilson

**Affiliations:** 1 Lancaster Environment Centre, Lancaster University, Lancaster, UK; 2 State Department for Crop Development & Agricultural Research, NARL Kabete, Waiyaki Way, Nairobi, Kenya; 3 Crop Bioscience Solutions Ltd., Arusha, Tanzania; 4 Pest Control Services, Ministry of Agriculture and Food Security, Arusha, Tanzania; 5 CABI, Nairobi, Kenya; 6 Centre for Ecology and Conservation, University of Exeter, Penryn, Cornwall UK

**Keywords:** Agroecology, Animal migration, Ecological epidemiology, Ecological modelling, Invasive species, Ecology

## Abstract

Invasive species have historically been a problem derived from global trade and transport. To aid in the control and management of these species, species distribution models (SDMs) have been used to help predict possible areas of expansion. Our focal organism, the African Armyworm (AAW), has historically been known as an important pest species in Africa, occurring at high larval densities and causing outbreaks that can cause enormous economic damage to staple crops. The goal of this study is to map the AAW’s present and potential distribution in three future scenarios for the region, and the potential global distribution if the species were to invade other territories, using 40 years of data on more than 700 larval outbreak reports from Kenya and Tanzania. The present distribution in East Africa coincides with its previously known distribution, as well as other areas of grassland and cropland, which are the host plants for this species. The different future climatic scenarios show broadly similar potential distributions in East Africa to the present day. The predicted global distribution shows areas where the AAW has already been reported, but also shows many potential areas in the Americas where, if transported, environmental conditions are suitable for AAW to thrive and where it could become an invasive species.

## Introduction

Global trade and transport have historically led to the movement of organisms, mostly for domestication, farming, etc. where they are in a controlled environment^1,2^. However, some movements of species are unintentional and can result in species becoming invasive in these new areas^3–5^. Invasive species, therefore, can produce massive economic and environmental damage due to their ability to spread without limitations^6–8^; and insects, being the most diverse group of organisms on Earth, are also one of the most invasive^9^. Some of the major problems caused by invasive insects include human disease vectors and agricultural and forest pests^10^, often impacting the health and economy of the countries affected^11^. Some well-known recent examples of invasive agricultural pests are the cotton bollworm, *Helicoverpa armigera* (Hübner), the diamondback moth, *Plutella xylostella* (Linnaeus), and the fall armyworm, *Spodoptera frugiperda* (J. E. Smith)^12–14^.

The African Armyworm (AAW) is the larval stage of the noctuid moth *Spodoptera exempta* (Walker, 1856). Like other armyworms^15^, AAW is considered a major pest species, historically the most important after locusts in parts of Africa^16,17^. AAW often occurs at high larval densities, causing outbreaks and, therefore, significant economic damage to crops and pasturelands^16,18^. The species is widely distributed across sub-Saharan Africa, where it especially affects Central, Eastern and Southern Africa, but the presence of the species has also been reported in Arabia, Southeast Asia, and Australia^19–21^. AAW caterpillars are a major pest of cereals and grasses, including some of the most economically important crops such as maize, rice or wheat^22^. Generally, low-density populations of the larvae persist throughout the continent, usually going unnoticed as they are in small numbers and have a cryptic coloration^23^. Many studies (e.g.^24–26^) have pointed out that it is after the first (short) rainy season in East Africa (around November or December) that the ‘primary’ (first) outbreaks occur. These outbreaks are caused by the mating and oviposition of the adult moths emerging from the low-density (dry season) populations, which are dispersed and scattered by the rainy season winds and end up concentrating in patchy areas where rainfall occurs^27,28^, that is thought to be due to convergent wind flows^23^. After these primary outbreaks, the long rainy season initiates a series of ‘secondary’ outbreaks, throughout eastern and central Africa, which may cause massive damage to crops, and can be monitored and predicted thanks to meteorological observation and monitoring^27,29,30^. In some countries, like Zambia, its maize production in 2012–2013 was reduced by 11% due to AAW attack^31^ and in 2017 it was estimated that 30–40% of the crop production could have been lost due to this pest^32^.

Since at least 1930, AAW outbreaks and moth trap data, as well as some meteorological data, have been collected in the most affected countries, including Kenya and Tanzania^16,21^. Subsequently, these data have been digitised and incorporated into data management and information systems, such as *WormBase*^33^, which was developed in the 1990s to aid in the prediction of AAW outbreaks. In the present study, we use forty years of AAW outbreak data to model the environmental suitability of the pest.

Species distribution models (SDMs) are modern tools that are used to characterize and predict the present and future distribution of a species, using species distribution data and environmental variables that affect, directly or indirectly, the species’ ecological niche or environmental suitability^34–36^. This provides a very useful tool for pest management activities, as it can help identify areas where the species might be present or vulnerable areas for the pest^37–39^. SDMs have been used to model the environmental suitability of other similar pest species, such as the fall armyworm, *S. frugiperda*, FAW, which is native to the Americas, but has recently invaded and spread throughout sub-Saharan Africa, into areas where the African armyworm is endemic^14^. This work was used to predict new areas in the world that could be suitable for FAW expansion, including parts of Asia and Oceania; predictions that have subsequently been realised (https://www.fao.org/fall-armyworm/monitoring-tools/faw-map/en/). Although the distribution of *S. exempta* in Africa and Arabia has been well established for at least 40 years^21^, and much is known about its feeding and migratory behaviour^16^, there is little information about its broader environmental requirements.

In this study, we generate the first predictive environmental suitability models for the African armyworm, using species distribution modelling techniques. We use occurrence data from reported larval outbreaks in Kenya and Tanzania, and variable selection methods to define the principal environmental variables that affect the geographical distribution of *S. exempta*. The generated models, which are local to Kenya and Tanzania, predict the present and future environmental suitability of the species under three different future-climate scenarios. For predicting the present suitability, we used the outbreak data from 1969 to 1990 and contrasted the generated model with the rest of the data, from 1991 to 2008. This meant we validated our model against data that are more independent than used in the majority of SDM studies, a highly recommended approach^40^. For the three future climate scenario models, we used all the outbreak data from Kenya and Tanzania, from 1969 to 2008 to forecast the 2061–2080 time period. We also model the global environmental suitability for the species by extrapolating these local data to the rest of the world to assess its invasion potential. Finally, we determine if models suggest that the African armyworm’s future distribution will likely intersect areas of cropland, which could demonstrate a need for preventive and control measures to target the vulnerable areas before they are attacked.

## Results

### Variable selection

**Figure 1:**
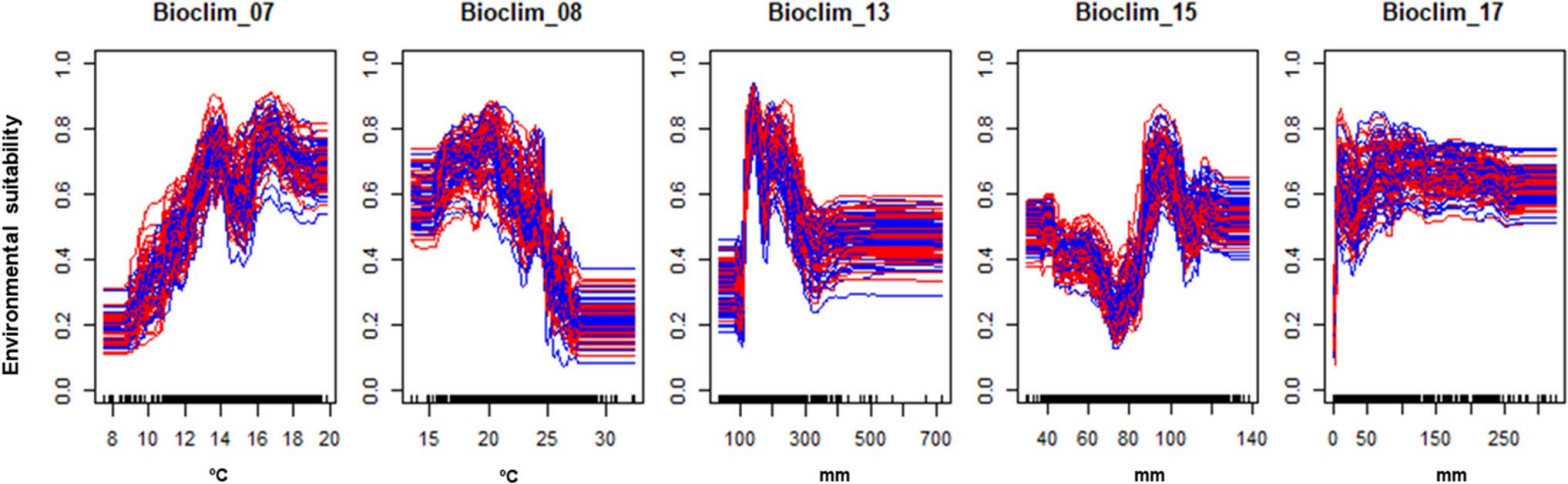
Response of *S. exempta* presences and absences to the selected variables. Bioclim_07 is temperature annual range, Bioclim_08 is mean temperature of the wettest quarter, Bioclim_13 is precipitation of the wettest month, Bioclim_15 is precipitation seasonality, and Bioclim_17 is precipitation of driest quarter.

The variable selection through PCA narrowed the environmental suitability components to five (Table 1). The variables are related to temperature and precipitation, and the AAW response to them can be seen in Fig. 1. Bioclim 07 (temperature range throughout the year) suggests that AAW do best in locations where the temperature variation is greater than around 12 °C annually. Variable Bioclim 08 is related to temperature during the wettest quarter and seems to suggest that AAW prefer temperatures between 15 and 25 °C during the rainy season, and anything greater than 25 °C is much less suitable. Variable Bioclim 15 is related to the seasonality of precipitation and suggests that AAW do best when rainfall varies by around 80–100 mm annually. Finally, Bioclim 13 and 17 are related to the amount of precipitation during the wet and dry season, respectively. During the wettest month, it seems to require a minimum of around 100 mm rain, but also seems to have a maximum of around 300 mm rain, above which it is less suitable, perhaps indicating its susceptibility to floods. During the driest quarter, it seems to be more versatile and can tolerate a wide range of precipitation, but there appears to be a minimum rainfall of around 10 mm, indicating that is also susceptible to drought.

**Table 1:** Variables selected by the PCA for the *S. exempta* environmental suitability models.

Variable name	Description
Bioclim 07	Temperature annual range
Bioclim 08	Mean temperature of the wettest quarter
Bioclim 13	Precipitation of the wettest month
Bioclim 15	Precipitation seasonality
Bioclim 17	Precipitation of driest quarter

### Model performance

The receiver operation characteristic (ROC) curve is a graphical way of illustrating the model’s ability to distinguish between binary classes at various threshold settings, and area under the curve (AUC) of the ROC is a value that measures the degree to which these classes can be distinguished between. This means that the closer to 1 the AUC value is, the better the model will be at separating classes, which in this case would be the environmental suitability of the species. AUC values of our models are considered to be ‘excellent’^41^, and TSS, values are considered ‘moderate’ and ‘substantial’^42^, therefore showing a good performance of the models, and that they are robust and accurate (Table 2). This indicates that the ecological suitability suggested by the generated models resemble the real probability of occurrence of the species, and therefore, its possible distribution.

**Table 2:** Internal evaluation statistics for the generated species distribution models (SDMs) generated.

Model	AUC	TSS
Predictive local model (1969–2000)	0.90 ± 0.01	0.62 ± 0.002
Present-time local all data model (1969–2008)	0.88 ± 0.02	0.59 ± 0.003
Present-time global all data model (1969–2008)	0.98 ± 0.03	0.99 ± 0.002

AUC and TSS values are average values ± standard deviation for the algorithms used in the SDMs.

### Environmental suitability of *S. exempta*

**Figure 2:**
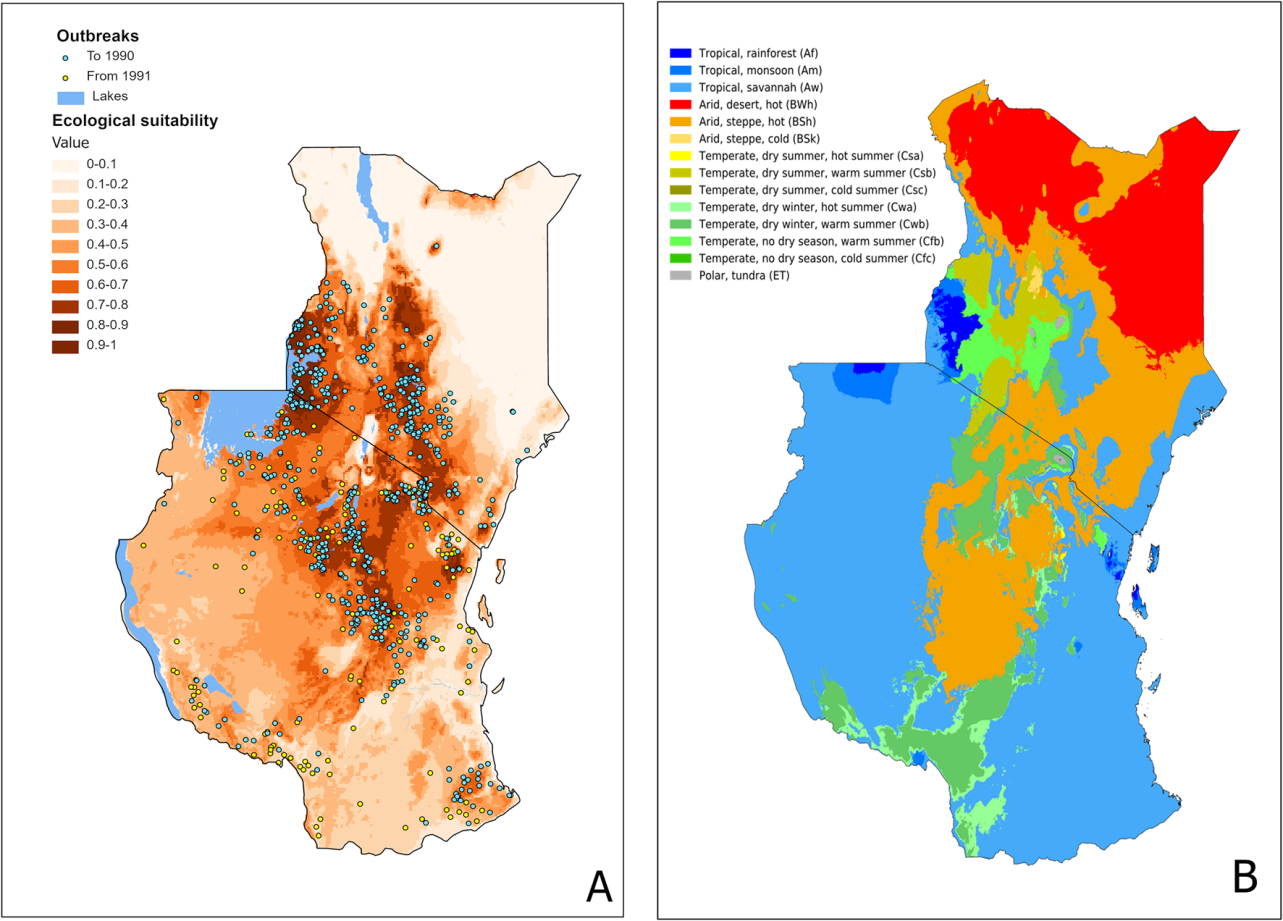
(**A**) *S. exempta* present-time environmental suitability model for Kenya and Tanzania. Points are the occurrence points from the outbreak data used for the models; (**B**) land cover map for Kenya and Tanzania (after Ref.^44^). Maps were generated in R v.4.0.2 104 (https://www.r-project.org/) using RStudio v.1.3.1093 (https://www.rstudio.com/).

Present-time environmental suitability models for the AAW in Kenya and Tanzania (Fig. 2A) show high suitability in the south and west of Kenya and the north and centre of Tanzania. These areas coincide with the occurrence points from the outbreak data used (blue dots in Fig. 2A); outbreaks are usually reported on crops such as maize, so it is likely that environmental suitability overlaps with agricultural land use. These suitable areas also coincide with sub-humid and tropical highlands; the paler or non-suitable areas coincide with more arid conditions, such as north-eastern Kenya^43^. Figure 2B shows a land use map extracted from Ref.^44^, indicating that the vegetation in the suitable areas of our model (Fig. 2A) are mainly grasslands, savannas and croplands. Regarding the prediction of the 1991–2008 outbreaks, all the points (yellow dots in Fig. 2A) seem to fall in areas with medium to high suitability, with AUC = 0.90, considered as ‘excellent’^41^, which indicates the model can accurately predict the areas that are suitable for outbreaks in the near future.

### Future and worldwide environmental suitability scenarios

**Figure 3:**
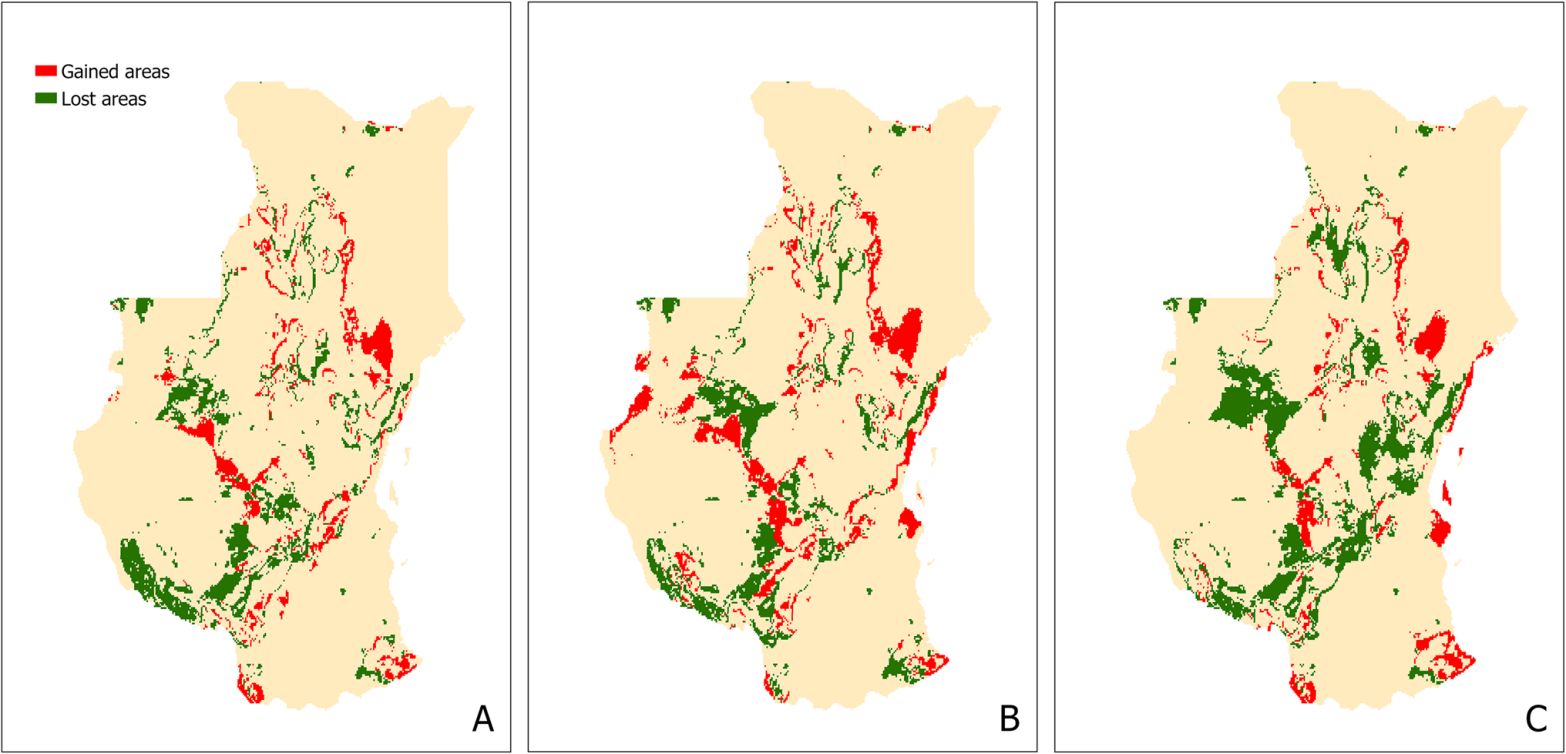
*S. exempta* future environmental suitability maps for Kenya and Tanzania for 3 different CO_2_ emission scenarios. (**A**) 2061-2080 SSP1-2.6, (**B**) 2061-2080 SSP3-7.0, (**C**) 2061-2080 SSP5-8.5. Gained areas are areas where the present-time model predicts as non-suitable, and the future-time model as suitable; lost areas are areas where the present-time model predicts as suitable, and the future-time model as non-suitable. Maps were generated in R v.4.0.2 104 (https://www.r-project.org/) using RStudio v.1.3.1093 (https://www.rstudio.com/).

Figure 3 presents three maps that show the difference in environmental suitability between present-time and three different CO_2_ emission scenarios between 2061 and 2080 in Kenya and Tanzania. The outputs of the three scenarios are very similar to each other. Scenario SSP1-2.6 (a gradual decline in CO_2_ emissions) show fewer gained areas (74,075 km^2^) than lost (109,500 km^2^), and the same happens with the extreme CO_2_ emission increase scenario—SSP5-8.5 (70,425 km^2^ of gained areas; 161,425 km^2^ of lost areas). Gained areas (109,625 km^2^) for scenario SSP3-7.0 (gradual increase in CO_2_ emissions), are however similar to the lost areas (106,350 km^2^). These results depict a future where the species seems to have a limited spread. Gained areas coincide mainly with cropland and grassland^45,46^. This all suggests that climate change might help the AAW distribution to expand and take over areas of grassland and cropland; but also limit its expansion in other areas where too many emissions might destroy these grasses and crops.

**Figure 4:**
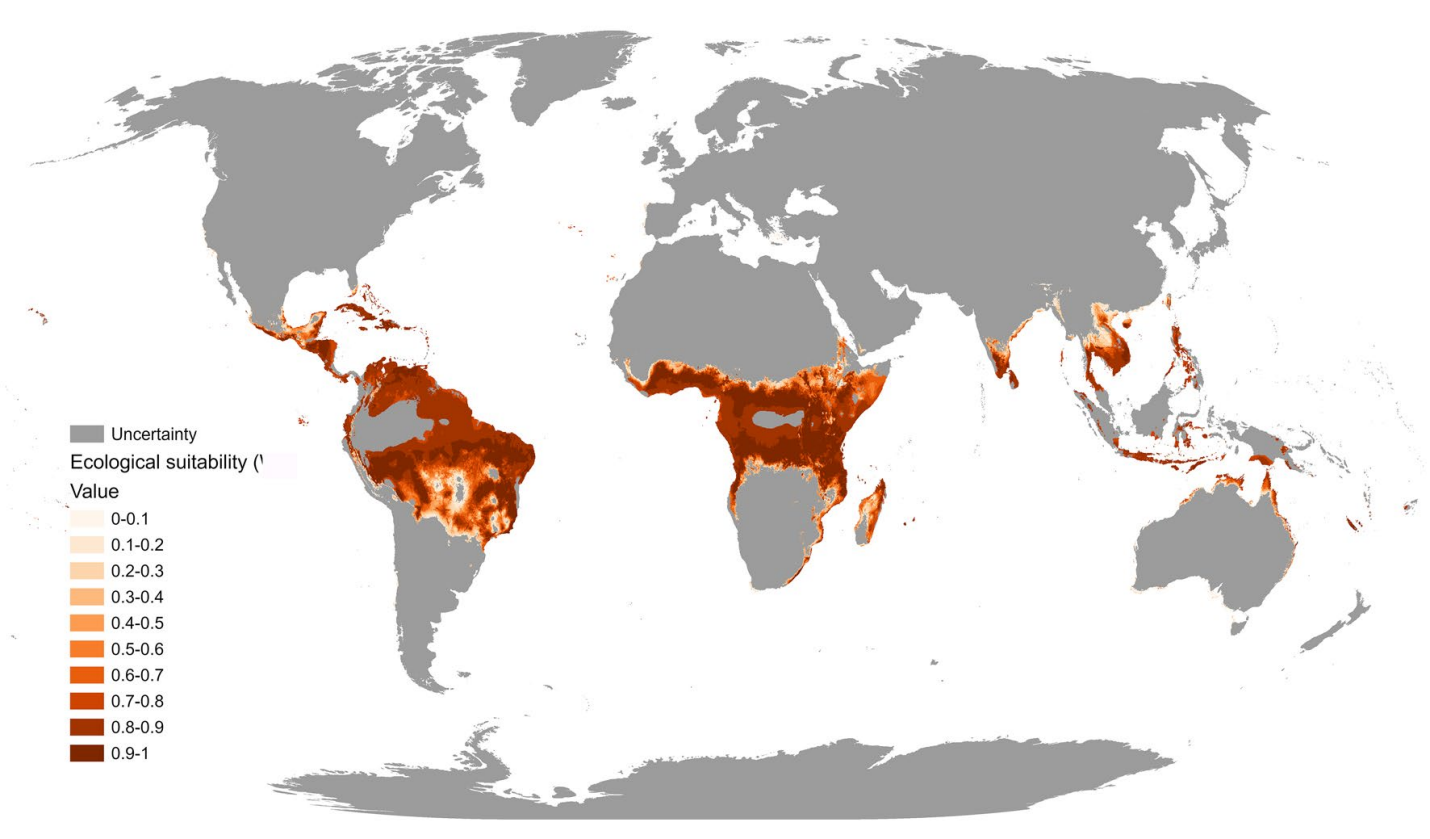
*S. exempta* present-time worldwide environmental suitability model. Grey areas represent uncertainty, calculated through MESS approach^47^. The map was generated in R v.4.0.2 104 (https://www.r-project.org/) using RStudio v.1.3.1093 (https://www.rstudio.com/).

The world environmental suitability model shows a marked high suitability in tropical areas, especially related to high, but not extreme, temperatures and precipitation (Fig. 4). It appears that the suitability overlaps the distribution of grasses, which is historically the main food source of the AAW, as it is noticeable in the Savannas, Pampas and Veldts, and seems to be delimited by arid areas and tropical deserts (e.g. Sahara, Kalahari, Atacama, etc.) as well as areas of extreme rainfall like rainforests (e.g. Amazon, Congo River Basin, South East Asia and Australian). However, as the models have only been constructed with climatic variables and not land use rasters, we cannot be completely certain that these forested areas could be suitable if converted to agriculture.

**Figure 5:**
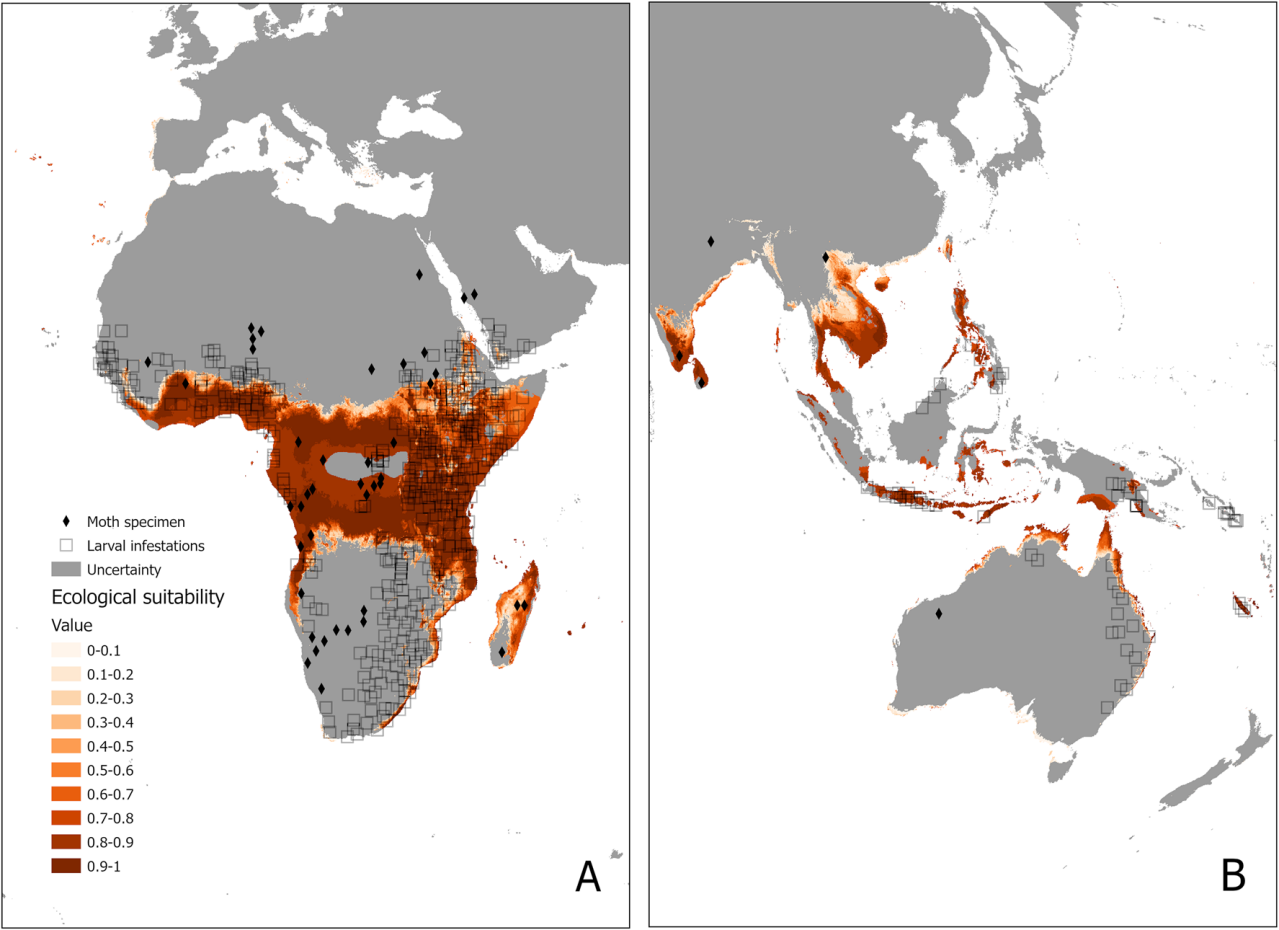
Recorded worldwide *S. exempta* larval infestations and moth specimens (reproduced with permission after Ref.^21^) overlapping Fig. 4 environmental suitability model. The map was generated in R v.4.0.2 104 (https://www.r-project.org/) using RStudio v.1.3.1093 (https://www.rstudio.com/).

When looking at the recorded distribution of AAW globally^21^ (Fig. 5), it very much resembles the world environmental suitability model (Fig. 4). Grey areas show where the projections are extrapolated outside of the climate conditions used to build the SDM, according to the results of the MESS approach^47^. Projections in these areas should be treated with extreme caution, as there is no way of knowing how accurate they are. In Africa, there is high suitability in the eastern, western, and central areas, where larval infestations have been recorded, even on the west of southern Africa. Madagascar is also predicted to be suitable for AAW outbreaks, although no larval infestations have been recorded there to our knowledge, but moth specimens have been found, indicating the possibility of being there. In Arabia, which has extensive larval infestations, only a limited area is predicted to be suitable, and with only medium suitability, probably due to it not being a very suitable climate, but in practice, irrigation could have permitted its viability and expansion. There is very high suitability in the west and south of India, and Sri Lanka (Figs. 4, 5), which coincides with the ghats where grasses are present, but the species has not yet been recorded there. Many AAW larval infestations and outbreaks have been reported in southern (but not northern) parts of Southeast Asia and the western Australian coast, coinciding with areas of medium to high suitability. With the exception of Hawaii^48^—where the model shows high suitability—the species has never been reported in the Americas. Nonetheless, the model does predict very high environmental suitability in some countries like Brazil, Colombia and Mexico (Fig. 4), which sets an alarm for its potential distribution and settlement if the species was to reach those areas. All this indicates that the model has been able to predict most of the actual worldwide distribution, using a database limited to a relatively small area in East Africa, and therefore, that it is a robust model.

## Discussion

In a world in which crop production often revolves around extensive monocultures, and global changes in climate and trade facilitate the spread of insect crop pests, there is increased potential for the introduction and spread of invasive species^49–51^. Understanding the environmental requirements of potentially invasive crop pests can identify areas at threat and facilitate targeted monitoring. Some authors have previously tried to do this by generating current or potential Species Distribution Models. Examples include important invasive pest species, such as the cotton bollworm, *H. armigera*, the diamondback moth, *P. xylostella*, the gypsy moth, *Lymantria* dispar (L.), the spotted wing drosophila, *Drosophila suzukii* (Matsamura), the European paper wasp, *Polistes dominula* (Christ), and the fall armyworm, *S. frugiperda*^12–14,52,53^. In this study we have constructed SDMs for the African armyworm, *S. exempta*, a pest endemic to sub-Saharan Africa. Our results identify those climatic variables that seem most important in determining the geographical distribution of AAW and provide a robust SDM for Kenya and Tanzania in the present time, as well as three different future climate change scenarios. We expand this to a predictive worldwide model that identifies areas, especially in the Americas and South Asia, where AAW has the potential to become invasive if it were introduced.

Selected variables for the environmental suitability of African armyworm outbreaks are mainly related to annual temperature variation and precipitation, especially during the wettest quarter, which is the rainy season. The rainy season plays an important role in the movement of AAW adults in Africa, as the winds that occur during it are key for the dispersal of the adult moths. Existing literature^23,26,29,54^ indicates that adult moths migrate along the dominant winds to grassland areas or crops, where they feed, causing subsequent larval outbreaks in nearby areas where they can disperse or migrate to. Precipitation outside the rainfall season is important for the low density populations of AAW that persist in these areas where outbreaks have occurred, during the dry season, as it stimulates the growth of grasses, providing the AAW with suitable habitats for feeding and breeding^55^, which could explain why variables like ‘precipitation seasonality’ or ‘precipitation of the driest quarter’ have been identified as important explanatory variables. Nonetheless, the areas where outbreaks occur (which we modelled) are not always the same as the ones where low-density populations settle (which we did not explicitly model). Temperature changes affect the species distribution too because, being ectotherms, their development and survival are temperature-dependent^56^.

The local present-time model depicts a robust environmental suitability for *S. exempta* in Kenya and Tanzania (Fig. 2A). Low environmental suitability coincides with arid or semi-arid areas, which may seem evident as extreme temperatures and dry conditions are not ideal for the development of its eggs and pupae^56,57^. Indeed, water and ambient humidity scarcity can affect the water balance of insects, impacting their survival, development and even their population dynamics, as seen in similar species, the FAW^58^. Climatic conditions in these areas can also affect its suitability indirectly. For example, changes in the water content and concentration of nitrogen and other minerals of the host plants, can negatively impact AAW adults’ fitness^59^. Additionally, plants that grow in arid or semi-arid areas are not suitable host plants of the AAW^16^, which mainly feeds on Graminae, and these require a certain level of humidity for their development. According to the generated model, sub-humid and tropical highlands are the most suitable areas for the AAW and, the known distribution of the AAW, besides the biology of the species, coincide with these areas. During the dry season, low-density armyworm populations are usually found in the highlands as the low temperatures extend their development^16^, which may explain why these tropical highlands are highly suitable. Looking at land cover and vegetation maps (e.g.^44,45^), the vegetation present in the suitable areas are mainly grasslands, savannas and croplands, which are the main host plants for the AAW.

The predictions of the environmental suitability for the 1991–2008 outbreaks (not included in the training dataset), appear to be accurate and robust, indicating that modelling present environmental suitability can be useful to predict outbreaks in the near future. These predictions can also be combined with population dynamic studies to predict outbreaks of the next few years, like other authors have previously done^30,60,61^.

Local future-scenario models (Fig. 3) are useful to predict where the species might be present in some years’ time. It is evident that climate change is altering the environmental conditions, therefore redesigning where species can live. It has been thoroughly documented that the distribution of many species is shifting to new areas, as well as disappearing from others^62–64^. This is especially important in pest management as predicting new areas could help set control measures for those areas and prevent outbreaks^39,65,66^. Although we produced models for three different CO_2_ emission scenarios, they all portray similar results, where there are suitable areas being both gained and lost. A positive side to this similarity in suitability is that management and control plans will probably be effective in all scenarios. On the other hand, it is interesting that such an aggressive pest like the AAW is predicted to show a slow expansion of their distribution, if compared to other similar pest species like processionary moths (*Thaumetopoea* spp*.*) or the box tree moth (*Cydalima perspectalis*)^67,68^. Climate change will likely alter the environmental suitability of all living organisms as it challenges their physiological limits^69^, and there is evidence that the geographical distribution of crop pests is moving increasingly polewards in response to climate change^70,71^. Due to this, it would be assumed that the expansion of the suitable areas would be much quicker or extensive, but these results might indicate the contrary, that climate change could reduce the suitable areas for its expansion. Factors affected by climate change, such as temperature, rainfall and relative humidity, seem to have mostly positive effects on fecundity and development of migratory pests like locusts^72,73^. However, for other lepidopteran pest species, like *H. armigera*, climate change has negatively affected its survival and reproduction^74,75^. Climate change is also reducing the amount of rainfall, which has had an impact on the ecosystem dynamics and vegetation structure of grasses in South Africa reducing grassland areas^76^, but also grass productivity, shifting these grasslands to shrubland and other tree-dominated biomes^77,78^. As grasses are the main food source for the AAW, it is coherent that all these lost suitable areas in our future scenario models might correspond to grass areas shifting to other vegetation patterns.

Global environmental suitability in the African continent resembles very much the previously reported distribution of African armyworm^21^ and appears in nearly all the same areas, that is, sub-humid areas, grasslands and croplands. Haggis’ study indicated that AAW has been recorded in India, South-East Asia, and Australia, where the models do predict a high environmental suitability, even though their presence there had not been used to generate it. This shows that the models are competent and can predict real areas where the species might expand into. There are areas, nevertheless, where the model does not predict high suitability, but the species has been recorded, like some parts of Indonesia, Arabia, and southern Africa. This could be due to the sample size and its limited geographic extent. Many authors (e.g.^79,80^) have reviewed this issue and it does seem to affect the accuracy and performance of SDMs. As our database is limited to Kenya and Tanzania, the selected variables will extrapolate to areas where the conditions are similar, that is why the prediction of suitability outside the tropics is not as accurate, as shown by the results of the MESS approach. Projections into colder regions seem likely to be inaccurate due to the variable response (Fig. 1), which have a clear upper limit. However, projections into areas with higher or lower precipitation rate might be more trustworthy due to a wider tolerance to change in precipitation^26^. Nonetheless, the worldwide model seems to predict an accurate environmental suitability in general.

In the global environmental suitability model, areas where the AAW has not been recorded but have a high suitability are intriguing. These are mostly in the Americas, especially between the tropics, where the climatic variables define the AAW’s niche. They also include coastal regions where there are grasses, like Pampas; or open woodlands, but also avoid tropical rainforests or arid areas due to their extreme conditions. The global environmental suitability of the AAW mirrors the environmental suitability and distribution of the FAW^14^ which has very similar environmental requirements, making them potentially competing species. The FAW, which is native to the American continent, was introduced into Africa, probably due to transportation of plants and crops, and rapidly spread to become one of the most important crop pests on the continent. Another example of this is *H. armigera*, which made a jump from Africa and Europe to the American continent^13^. The global model suggests that a similar thing could happen with the AAW on the American continent if it were introduced. Countries like Brazil, which is one of the world’s biggest maize producing countries could, in time, become hotspots for the AAW and enhance this global problem. Our models, and the variables used however, do not consider anthropogenic factors that could increase the migration and dispersal of *S. exempta*, such as global connectivity and human-mediated transport^81^, as it has been done for the fall armyworm^14^. If considered in future studies, this could confirm our findings about *S. exempta* ability to disperse throughout the American continents, which has already been considered as a potential risk^82^. This manifests the importance of revisiting and tightening international agricultural biosecurity, as invasive species are transported to new territories in a daily basis, aggravating the problem^83,84^.

Characterizing the climatic variables that explain or delineate the AAWs niche will help with a better understanding of the species’ biology and its possible management^85^. Future and global scenario models based on climatic variables, like the ones used in this study, are important to understand how invasive pest species might react to climate change or new areas if they are transported there. In fact, IPM studies often use these SDMs and niche characterization^86^ of important pest species such as the fall armyworm, *S. frugiperda*^15^, underlying its importance. However, to understand how the species will disperse in space and time, models should be used as part of a bigger research effort, including natural competence, or anthropogenic factors, such as bias in outbreak reporting, land use and management, transport, etc.

Finally, it is worth noting that SDMs are generally only used to predict suitable abiotic environments and seldom include detailed information regarding the presence of potential competitor species or natural enemies. Invasive fall armyworms have rapidly expanded throughout the African continent and globally^88^. It is considered a very aggressive and cannibalistic alien pest^89,90^ and feeds on a range of plant species, including the cereals and grasses that AAW specialises in, meaning there is a possibility of displacement, as it appears to be doing with other sympatric species, such as the Asiatic pink stem borer, *Sesamia inferens* (Walker) or the maize stalk borer, *Busseola fusca* (Füller)^91,92^. Given this, it is possible that although our SDM suggests that parts of the Americas are environmentally suitable for AAW to invade, in this environment it would be potentially competing with the native FAW, which is much more aggressive than AAW and is likely to be the stronger intra-guild competitor. It is therefore possible that AAW has previously reached the Americas but has failed to establish there due to competitive interactions with FAW or other natural enemies.

## Materials and methods

### Distribution data compilation

The presence records for Kenya and Tanzania were obtained from an updated version of *WormBase*^33^, which is a data management and information system that includes AAW outbreak and trap data for both countries since 1969. Outbreak data were used for the present study, where only presence records with defined geographic coordinates, following the WGS84 geographic coordinate system were used. Presence points that were inaccurate and duplicates were filtered using ArcGIS Pro. In total, 721 occurrence points, from 1984 to 2008, were obtained. 568 occurrence points were recorded from the years 1969–1990, and were used to make the first model, which predicted the current distribution.

### Environmental data

Species Distribution Models (SDMs) require selecting biotic and/or abiotic environmental variables that relate to the distribution of the modelled species^40^, and to minimize uncertainties in modelling predictions it is important to understand which variables are more significant to the species by performing a good variable selection^93^.

Variables used in this study were the WorldClim Version 2^94^ bundle of 19 global climatic layers from 1970 to 2000 in a 5 × 5 km resolution; and WorldClim CMIP Phase 6 (CIMP6)^95^ global climatic layers for future suitability models. We selected the 2061–2080 period for the BCC-CSM2-MR General Circulation Model (GCM)^96^ and three Shared Socio-economic Pathway (SSP): SSP1-2.6, which shows a gradual decline in emissions; SSP3-7.0, which would be an intermediate scenario where the CO_2_ emissions continue to rise in a similar fashion to now; and SSP5-8.5, which shows a dramatic rise in CO_2_ emissions^97^.

#### Variable selection

In previous modelling studies for the fall armyworm^14^, the variable selection was based on the life-history and environmental requirements for the species. Nonetheless, other studies^98–100^ suggest other analyses, such as Ecological Niche Factor Analysis (ENFA) or Principal Component Analysis (PCA), may be more robust, as they result in uncorrelated variables. This both eliminates information that might be redundant and means that the forecasts are not affected by changes in the correlation between environmental variables between time periods or regions. We followed the methodology described by Gómez-Undiano, 2018^100^, a method derived from Petipierre et al.^98^, which showed that a PCA resulted in a more accurate variable selection for better models. Therefore, we did a PCA with all the previously chosen variables and reduced the number to some main ones, based on the variance explained in the presences of *S. exempta*; this being the variables that had the greatest loadings on some of the PCA axes. The variables used for the future predicted suitability were the same as the ones resulting in the PCA, but from the 2021–2040 bundle. The variable selection was carried out in R v.4.0.2^101^ using RStudio v.1.3.1093.

### Modelling environmental suitability

SDMs can be generated only with presence points but this can result in inaccurate and biased models^102^, so often, absence points are used too. However, absences are difficult to obtain, especially for mobile species like insects. However, studies suggest that selecting pseudo-absences, which could be generated randomly, helps to improve the quality of the models and their accuracy^102–104^. We followed the BIOMOD modelling algorithm^105^, using the ‘biomod2’ package^106^ in R for pseudo-absence generation, and selected 700 pseudo-absence points for the local distribution models in Kenya and Tanzania, to match the number of occurrences^104^. When extrapolating pseudo-absence data to the rest of the World, some authors^107,108^ suggest delimiting a geographical background to which the species could reasonably disperse, can improve SDM. We generated a background area (for the Worldwide ensemble model) of the limited area of Kenya and Tanzania to reduce extrapolation of the variables to non-analogue areas.

Predicting global suitability from a limited area, such as Kenya and Tanzania, means that predictions could be extrapolated to areas with very different climate to Kenya and Tanzania, which could be highly erroneous. To ensure the predictions are only made in areas with conditions similar to those in the data used to construct SDMs, the Multivariate Environmental Similarity Surface (MESS)^47^ was calculated using the R package ‘dismo’^109^.

Choosing one modelling statistic method can be challenging because different methods have advantages and disadvantages and tend to produce variable predictions. However, ensemble modelling results in producing more robust and reliable models^110,111^. We created an ensemble that includes five algorithms based on logistic regression and machine learning: artificial neural networks (ANN), classification tree analysis (CTA), flexible discriminant analysis (FDA), generalised additive models (GAM), generalised linear models (GLM), MaxEnt, random forest (RF) and Surface Range Model (or BIOCLIM). This process was undertaken using default parameters from the ‘biomod2’ package in R.

To evaluate the accuracy and robustness of the ensembled models, internal validation, which is included by default in the ‘biomod2’ setting, was used. We split the distribution data randomly into two, with 70% being used for the SDM calibration and 30% the validation set, using the area under the curve (AUC) of the receiver operation characteristic (ROC), and true skill statistic (TSS). 100 replicas were generated for each algorithm used, and models for which validation with AUC > 0.7 or TSS > 0.6 were selected to generate the final ensembles. Although studies generally use a 70–30% data split for the training and testing data e.g.^14,112^, we also generated additional models with different data-splits (10, 20, 30, 40, 50, 60, 80 and 90%) to ensure the model validation was robust (Supplementary Materials). External validation of the predictive model was constructed using outbreak data from 1969 to 1990 was also performed, by calculating the AUC of the model against the outbreak points from 1991 to 2008 as the validation set.

In total, three ensemble models showing environmental suitability for *S. exempta* were generated: (1) a predictive local model using recent (1970–2000) environmental conditions for Kenya and Tanzania and outbreak data sub-sample from years 1969 to 1990, which was validated against more recent data (1991–2008); (2) a present-time local model for Kenya and Tanzania using all outbreak data (1969 to 2008) with three projections for three CO_2_ emission scenarios (A. SSP1-2.6; B. SSP3-7.0; and C. SSP5-8.5) between 2061 and 2080; and, (3) a Worldwide present-time model using all outbreak data (1969 to 2008).

When looking at the future-scenario models, it is sometimes difficult to determine which are new areas that are more or less suitable for *S. exempta*. To make it easier to visualise, we converted the future scenario model projections and the present time model (using all the outbreak data) into binary maps using the cut-off values, based on TSS, of each projection. Then we combined each future scenario model projection with the present time one to get a categorical map showing new suitable and non-suitable areas.

## Supplementary Information

Supplementary Legends.

Supplementary Figure S1.

## Data Availability

The datasets generated during and/or analysed during the current study will be available in the DRYAD repository, after the manuscript is accepted [https://datadryad.org/stash/share/t-EgQOweHgcOHQ_paK1ao6PQuRsnjkGCSh63_HD4n00] with DOI number [10.5061/dryad.sbcc2fr9b].
