# Author Correction: Predicting potential global and future distributions of the African armyworm (*Spodoptera exempta*) using species distribution models

**DOI:** 10.1038/s41598-022-24222-5

**Published:** 2022-11-18

**Authors:** Irene Gómez-Undiano, Francis Musavi, Wilfred L. Mushobozi, Grace M. David, Roger Day, Regan Early, Kenneth Wilson

**Affiliations:** 1 Lancaster Environment Centre, Lancaster University, Lancaster, UK; 2 State Department for Crop Development & Agricultural Research, NARL Kabete, Waiyaki Way, Nairobi, Kenya; 3 Crop Bioscience Solutions Ltd., Arusha, Tanzania; 4 Pest Control Services, Ministry of Agriculture and Food Security, Arusha, Tanzania; 5 CABI, Nairobi, Kenya; 6 Centre for Ecology and Conservation, University of Exeter, Penryn, Cornwall UK

Correction to: Correction to: *Scientific Reports* https://doi.org/10.1038/s41598-022-19983-y, published online 28 September 2022

The original version of this Article contained an error in the legend of Figure 3.

“**Figure 3**. *S. exempta* future environmental suitability maps for Kenya and Tanzania for 3 different CO_2_ emission scenarios and two different time periods. (**A**) 2021-2040 SSP1-2.6, (**B**) 2021-2040 SSP3-7.0, (**C**) 2021–2040 SSP5-8.5, (**D**) 2061-2080 SSP1-2.6, (**E**) 2061-2080 SSP3-7.0, and (**F**) 2061-2080 SSP5-8.5.”

now reads:

“**Figure 3**. *S. exempta* future environmental suitability maps for Kenya and Tanzania for 3 different CO_2_ emission scenarios. (**A**) 2061-2080 SSP1-2.6, (**B**) 2061-2080 SSP3-7.0, and (**C**) 2061-2080 SSP5-8.5.”

The original Article has been corrected.

