# Supplementary material for: Predicting potential global and future distributions of the African armyworm (Spodoptera exempta) using species distribution models
Source: Sci Rep. 2022 Sep 28;12:16234. doi: 10.1038/s41598-022-19983-y (PMC9519994; doi:10.1038/s41598-022-19983-y)
Supplement: Supplementary file 1 — Supplementary Legends. [file 41598_2022_19983_MOESM1_ESM.docx]

**Supplementary material**

**Figure S1.** Effect of different data splits on ensemble SDM projections for *S. exempta* present-time environmental suitability model for Kenya and Tanzania (figure 2). **A)** 10%, **B)** 20, **C)** 30, **D)** 40, **E)** 50, **F)** 60, **G)** 80 and **H)** 90%. Maps were generated in R v.4.0.2 104 (https://www.r-project.org/) using RStudio v.1.3.1093 (https://www.rstudio.com/).
