# Supplementary figures and images for: Predicting potential global and future distributions of the African armyworm (Spodoptera exempta) using species distribution models
Source: Sci Rep. 2022 Sep 28;12:16234. doi: 10.1038/s41598-022-19983-y (PMC9519994; doi:10.1038/s41598-022-19983-y)

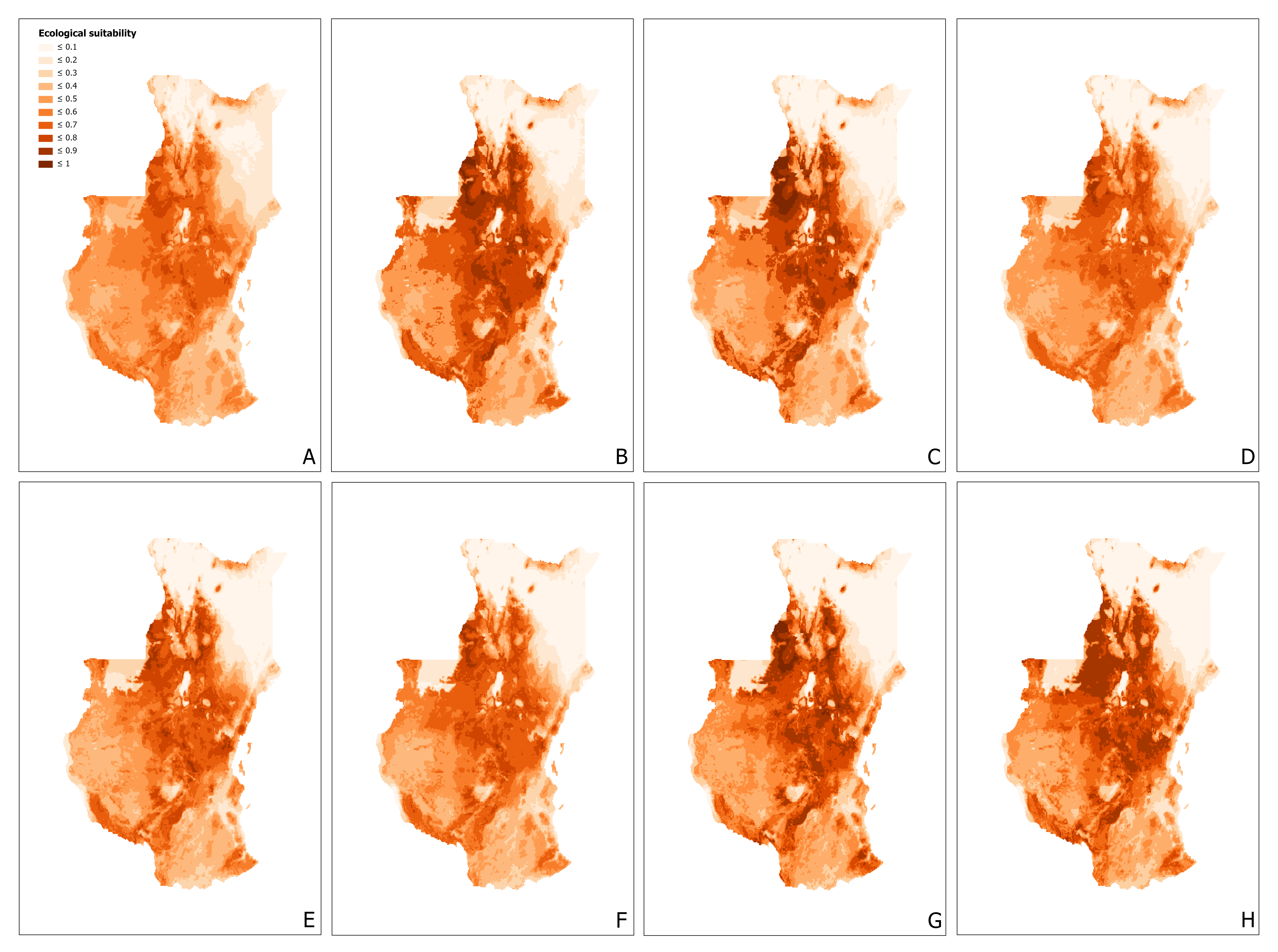

Supplement: Supplementary file 2 — Supplementary Figure S1. [file 41598_2022_19983_MOESM2_ESM.tif]
